# Factors Influencing Treatment Success in Cholesteatoma Management: A Cross-Sectional Study

**DOI:** 10.3390/jcm13092606

**Published:** 2024-04-29

**Authors:** Sarah Alshehri, Mohammed Abdullah M. Al Shalwan, Abdulkhaliq Abdullah A. Oraydan, Abdulrahman Saeed H. Almuaddi, Ahmed Jubran A. Alghanim

**Affiliations:** 1 Otology and Neurotology, Department of Surgery, College of Medicine, King Khalid University, Abha 61423, Saudi Arabia; 2 College of Medicine, King Khalid University, Abha 61423, Saudi Arabia; 438802269@kku.edu.sa (M.A.M.A.S.); 438802257@kku.edu.sa (A.A.A.O.); 438802270@kku.edu.sa (A.S.H.A.); 438802278@kku.edu.sa (A.J.A.A.)

**Keywords:** cholesteatoma, treatment outcomes, preoperative imaging, surgical approaches

## Abstract

**Background/Objectives:** Cholesteatoma presents significant management challenges in otolaryngology. This study aimed to delineate the influence of demographic and clinical characteristics, preoperative imaging, and surgical approaches on treatment success in cholesteatoma management. **Methods:** A cross-sectional analytical study was conducted at the Otolaryngology Department of the University Hospital from January 2021 to December 2022. It included 68 patients diagnosed with cholesteatoma, focusing on three objectives: assessing the impact of demographic and clinical characteristics on treatment outcomes, evaluating the predictive value of preoperative imaging findings, and analyzing the influence of surgical factors. **Results:** The study population predominantly consisted of male (56%) and Saudi (81%) patients, with an average age of 45 years. Logistic regression revealed that older age (OR: 1.05), male gender (OR: 0.63), and non-Saudi Arab ethnicity (OR: 2.14) significantly impacted treatment outcomes. Clinical characteristics such as severe disease severity (OR: 3.00) and longer symptom duration (OR: 0.96) also influenced treatment success. In preoperative imaging, labyrinthine fistula (Regression Coefficient: 0.63) and epidural extension (Coefficient: 0.55) emerged as key predictors. The surgical factors that significantly affected the outcomes included the extent of surgery (Complete Removal OR: 3.32) and the use of endoscopic approaches (OR: 1.42). **Conclusions:** This study highlights that patient demographics, clinical profiles, specific preoperative imaging features, and surgical strategies multifactorially determine cholesteatoma treatment success. These findings suggest the necessity for a tailored approach in cholesteatoma management, reinforcing the importance of individualized treatment plans based on comprehensive preoperative assessments.

## 1. Introduction

Cholesteatoma, a destructive and expanding growth in the middle ear and mastoid, presents unique challenges in otology [1]. Characterized by the accumulation of keratinizing squamous epithelium, this pathology can lead to a wide range of complications, from hearing loss to life-threatening infections [2]. Despite advancements in medical imaging and surgical techniques, the management of cholesteatoma remains a subject of ongoing research due to its recurrent nature and the potential for residual disease post-treatment [3].

The complexity of cholesteatoma arises from its varied presentations and the intricacies of its pathophysiology [4]. Several factors contribute to its development, including Eustachian tube dysfunction, chronic otitis media, and genetic predisposition [5]. The heterogeneity of the disease manifests in its clinical course, with some patients experiencing aggressive growths that rapidly lead to complications, while others may have indolent lesions with minimal symptoms [6]. The treatment of cholesteatoma typically involves surgical intervention, with approaches ranging from canal wall-up to canal wall-down mastoidectomy, often complemented by tympanoplasty for hearing restoration [7]. However, the success of these interventions is not solely dependent on the removal of the cholestatic sac but also on the patient’s demographic and clinical profile, preoperative imaging findings, and the specific surgical techniques employed [7,8].

The literature reflects a spectrum of perspectives on the factors influencing the outcomes of cholesteatoma surgery [9]. Age, gender, and ethnicity have been variably associated with treatment results, but the extent to which these demographic factors consistently affect prognosis remains unclear [10]. Additionally, clinical characteristics such as the severity of disease and duration of symptoms before intervention are believed to impact success, suggesting a possible advantage for early diagnosis and treatment [11]. Preoperative imaging, particularly high-resolution computed tomography (CT) and magnetic resonance imaging (MRI), has improved the preoperative assessment of cholesteatoma [12,13]. Imaging findings, such as the presence of mastoid air cell opacity or ossicular chain erosion, can provide invaluable information for surgical planning [13,14]. Yet, the predictive value of these imaging characteristics in the context of treatment success has not been fully established [15]. Surgical factors, including the choice of surgical approach and the extent of surgery, have been debated for their roles in patient outcomes [8,16]. The use of adjunct procedures such as ossiculoplasty and mastoid obliteration, the implementation of intraoperative monitoring, and the initiation of postoperative rehabilitative measures further add layers of complexity to outcome prediction [17]. Nevertheless, evidence for the systematic influence of these variables on the postoperative prognosis is still being consolidated [17].

Thus, the research gap lies in the comprehensive analysis of these multifactorial influences on the success of cholesteatoma treatment. The objectives are as follows: (1) to assess the association between demographic and clinical characteristics of patients diagnosed with cholesteatoma and their treatment outcomes; (2) to evaluate the impact of preoperative imaging findings on treatment success and their predictive value; and (3) to analyze surgical factors, including the chosen surgical approach, extent of surgery, and use of adjunct procedures, to delineate their influence on postoperative outcomes. The alternate hypothesis proposes that specific demographic characteristics (such as age and ethnicity), clinical profiles (including disease severity and symptom duration), distinct preoperative imaging findings, and particular surgical approaches are significantly associated with the success of cholesteatoma treatment.

## 2. Materials and Methods

### 2.1. Study Design and Ethics

This research was a hospital-based, cross-sectional analytical study conducted at the Otolaryngology Department of the University Hospital. This study spanned from January 2021 to December 2022, enrolling patients diagnosed with cholesteatoma based on clinical examination, audiological assessment, and radiological confirmation through high-resolution computed tomography (CT). Ethical approval for this study was obtained from the Institutional Review Board (IRB) of the University Hospital, under reference number REC-ENT-136/1345 on 23 December 2021. This study was conducted in full accordance with the ethical principles of the Declaration of Helsinki and local regulations. Written informed consent was acquired from all participants after a full explanation of this study’s purpose, the nature of the procedures involved, and the potential risks and benefits.

### 2.2. Participants

The participants of this study were individuals diagnosed with primary or recurrent cholesteatoma who underwent surgical intervention at our tertiary care facility. The inclusion criteria for this study were as follows: patients must be 18 years or older, diagnosed with primary or recurrent cholesteatoma as confirmed by otoscopic examination and high-resolution computed tomography (CT) [12], and must have undergone surgical treatment with at least one year of follow-up post-surgery. The exclusion criteria included patients under 18 years of age, those who did not undergo surgical treatment, those with less than one year of postoperative follow-up, patients with concurrent ear pathologies that could independently influence surgical outcomes such as autoimmune inner ear diseases [15], patients who had undergone otological surgery for conditions other than cholesteatoma within six months prior to the study period, and patients with concurrent neoplastic diseases of the ear. Recurrent cases were defined as those with re-emergence of cholesteatoma after a previous surgical attempt for removal, as confirmed by postoperative follow-up and imaging [12,15].

To provide a clear understanding of our participants’ surgical histories, we classified previous surgeries into three main types: tympanoplasty, which involves reconstruction of the eardrum and middle ear structures; mastoidectomy, aimed at removing infected air cells within the mastoidal bone, a common procedure for managing chronic otitis media and its complications including cholesteatoma; and ossiculoplasty, which is performed to repair or replace the small bones in the middle ear. These details are crucial for assessing the influence of past surgical interventions on the outcomes of the current treatment.

In our study, the patient data were systematically collected at various treatment stages. The preoperative details, including demographics and imaging findings, were obtained from records post-diagnosis and surgical planning. The surgical team meticulously documented intraoperative data, such as surgical approach and procedure extent. Postoperative information, including treatment outcomes and complications, was gathered during follow-ups at one, three, six, and twelve months. This structured methodology ensured comprehensive data capture throughout patient care in cholesteatoma management. We combined electronic records, patient interviews, and clinical assessments for a thorough analysis. To ensure data integrity, we implemented a robust protocol for resolving discrepancies, involving cross-verification, senior clinician review, and consultation of source documents, with necessary patient re-consultations. All resolved discrepancies were documented in detail, and regular audits, along with training on standard procedures, were conducted to maintain data quality.

### 2.3. Clinical Characteristics

Data on disease severity, duration of symptoms, and history of prior surgeries were collected from a detailed review of patient medical records, including surgical notes and otological examinations. The disease severity was categorized based on intraoperative findings, supplemented by preoperative imaging reports. The duration of symptoms was established based on patient-reported onset of symptoms. The disease severity was assessed using a combination of clinical presentation and radiological findings. Mild cases were confined to the epitympanum with no ossicular or mastoid involvement, moderate cases involved ossicular erosion or mastoid air cell opacity without inner ear or facial nerve complications, and severe cases showed extensive disease with labyrinthine fistulae, facial nerve involvement, or intracranial extensions. The clinical categorization was supplemented by detailed radiological findings from preoperative high-resolution CT and, when available, magnetic resonance imaging (MRI) scans. Discrepancies in severity assessment were resolved through multidisciplinary discussions involving otolaryngology surgeons and neuroradiologists. Hearing loss was classified according to pure-tone audiometry thresholds: mild hearing loss was defined as 26–40 dB, moderate as 41–55 dB, and moderate/severe as greater than 55 dB, following the guidelines set by the World Health Organization [18].

Facial nerve involvement is defined as any degree of facial nerve dysfunction resulting from the cholesteatoma or its treatment, assessed using the House–Brackmann grading system [19]. This includes Grade II (slight dysfunction) to Grade VI (total paralysis), providing a standardized measure of facial nerve function [19].

### 2.4. Preoperative Imaging

In this study, the assessment of facial nerve involvement was approached through a combination of radiological imaging and clinical evaluation. Prior to surgery, high-resolution computed tomography (CT) and magnetic resonance imaging (MRI) were utilized to detect anatomical changes indicative of potential facial nerve compromise, such as bony erosions, the presence of a cholesteatoma near the facial nerve canal, or inflammatory changes. These imaging findings provided a preliminary indication of potential risks to the facial nerve. Postoperatively, the functional status of the facial nerve was evaluated using the House–Brackmann classification system, which assesses the degree of facial nerve paralysis based on observable clinical symptoms. This comprehensive approach allowed for a detailed assessment of both the anatomical and functional aspects of facial nerve involvement.

The radiological data in our study were derived from preoperative CT and MRI scans. Two independent radiologists assessed these to identify cholesteatoma-related features, including mastoid air cell opacity, ossicular chain erosion, epidural extension, labyrinthine fistula, and facial nerve involvement. Each radiologist conducted an initial independent review and documented their findings. Discrepancies between their assessments were identified through comparison and addressed in consensus meetings involving both radiologists and a senior expert in otolaryngology imaging. These meetings facilitated open discussion and re-evaluation of divergent interpretations. In instances in which consensus was not reached, the senior radiologist provided an adjudication. To ensure accuracy and consistency from the beginning, we also held regular training and calibration sessions for the radiologists, focusing on aligning interpretation criteria. The meticulous documentation of the outcomes of these meetings and the emphasis on continuous radiologist training contributed significantly to the integrity and reliability of our radiological data. In our study, the surgical management of cholesteatoma was executed using either endoscopic or microscopic approaches, with the choice between these two primarily based on the disease’s extent, location, patient anatomy, and surgeon expertise. The endoscopic technique, advantageous for its minimal invasiveness and superior visualization, was typically employed for less extensive cases confined to the middle ear, facilitating precise removal of cholesteatoma while preserving the ossicular chain. Conversely, the microscopic approach, offering a broader field of view, was used in more extensive cases, involving significant mastoid involvement or complex reconstructions. This method often involved a post-auricular incision and a canal wall-up or -down mastoidectomy. Adjunct procedures utilized in this study include ossiculoplasty, which involves the reconstruction or replacement of the ossicles to restore auditory function; mastoid obliteration, aimed at filling the mastoid cavity with bioactive materials to prevent reformation of cholesteatoma and facilitate postoperative management; and tympanoplasty, which repairs or reconstructs the tympanic membrane to improve auditory function and seal perforations.

The selection between these techniques introduced variability in surgical strategies and execution due to differing surgeon experiences and comfort levels with each method. The surgeons’ preferences, based on their expertise and familiarity with either endoscopic or microscopic procedures, led to distinct decision-making processes and potentially diverse treatment choices for similar clinical scenarios. Surgical interventions were categorized as either microscopic or endoscopic, with no cases employing a combined approach during the study period. Despite aiming for standardized protocols, inherent differences in surgical styles, techniques, and intraoperative decision-making among surgeons remained a challenge. These variations encompassed aspects like handling surgical instruments and approaches to reconstruction, underlining the complexity of standardizing surgical methods in cholesteatoma treatment. This study thus aimed to balance complete disease eradication with ear function preservation, ensuring effective, personalized treatment while acknowledging the inherent challenges in standardizing surgical approaches. Intraoperative monitoring included facial nerve monitoring to prevent nerve damage and continuous audiometric monitoring during ossicular chain manipulation to preserve hearing.

### 2.5. Surgical Technique and Monitoring

In the surgical approach, continuous audiometric monitoring was performed using a Nerve Integrity Monitor (NIM) system. This system was specifically utilized to provide real-time auditory feedback during ossicular chain manipulation. It operates by delivering continuous pure-tone audiometry at critical speech recognition frequencies (500 Hz, 1000 Hz, 2000 Hz, and 4000 Hz). Electrodes were strategically placed on the promontory of the middle ear to monitor changes in the amplitude and latency of auditory responses.

In this study, endoscopic surgical approaches were primarily utilized for less extensive cholesteatoma cases confined to the middle ear and epitympanic areas without significant bone destruction. This method’s success in these instances was marked by its minimal invasiveness and superior visualization capabilities within restricted spaces. However, it is important to note that, for more severe cases featuring extradural extensions or labyrinthine fistulas, a microscopic approach or a combination of both endoscopic and microscopic techniques was employed. These complex scenarios required the broader access and more extensive tissue manipulation provided by the microscopic technique, which are less feasible with endoscopy alone. This distinction in surgical approach selection based on disease severity and anatomical involvement ensures appropriate management tailored to specific clinical conditions.

### 2.6. Outcome Assessment and Data Verification Strategies

In this study, the primary outcome was explicitly defined as the absence of residual or recurrent cholesteatoma, confirmed through postoperative imaging—either CT or MRI—at the one-year follow-up mark. This measure was specifically chosen to directly evaluate the success of the surgical intervention and its effectiveness in completely eradicating cholesteatoma, which is crucial for determining long-term treatment success. Complementing this, the secondary outcomes included the improvement or stabilization of hearing levels, as measured by pure-tone audiometry, along with the reduction of clinical symptoms such as otorrhea and hearing loss. Furthermore, any postoperative complications were noted from follow-up clinical records. For patients who underwent subsequent surgeries, assessments were consistently made based on their most recent procedure to ensure comparability across the study group. This methodology ensured that each participant’s data accurately reflected their current treatment status, providing a uniform and cohesive basis for analysis. In instances in which the data were sourced differently—such as from patient interviews versus electronic records—cross-verification procedures were implemented to ensure consistency and reliability. Additionally, the postoperative rehabilitation protocols were individually tailored, incorporating audiometric follow-ups, vestibular rehabilitation as needed, exercises for eustachian tube dysfunction, and, where necessary, hearing aid fittings.

A successful treatment outcome is defined by the absence of disease recurrence, as confirmed by otoscopic and radiographic examination, improvement or stabilization of hearing levels measured by audiometry, reduction in clinical symptoms such as otorrhea and hearing loss, and the absence of major postoperative complications, such as facial nerve paralysis or severe infection.

### 2.7. Sample Size Calculation

In determining the sample size for our study on cholesteatoma treatment success, we utilized G*Power statistical software (3.1 Version), targeting a medium effect size of 0.4. This decision reflects the expected clinical variability and outcome differences in cholesteatoma management. With parameters set at an effect size of 0.4, an α error probability of 0.05, and a power of 0.8, the analysis recommended a minimum sample size of 68 participants. This number is deemed sufficient to detect a medium effect size with 80% power and a 5% alpha level, offering a pragmatic balance between statistical validity and the practicalities of studying a relatively rare condition.

### 2.8. Data Analysis

In this study, data analysis was conducted using IBM SPSS Statistics Version 24, adhering to stringent preprocessing for accuracy and consistency. The Kolmogorov–Smirnov test confirmed the normal distribution of the data, validating the use of parametric tests for analysis. In addressing the study’s objectives, tailored statistical methods were meticulously chosen to ensure analytical precision. Multivariate logistic regression was utilized for Objective 1, assessing the relationship between demographic and clinical characteristics and treatment outcomes, due to its efficacy in handling multiple predictors and a binary outcome. Objective 2, focusing on the impact of preoperative imaging findings, employed Pearson’s correlation for evaluating the strength and direction of relationships, followed by linear regression to ascertain the predictive values of these imaging features. For Objective 3, examining surgical factors’ influence on outcomes, analysis of variance (ANOVA) was applied to detect differences in treatment success across surgical approaches, supplemented by post hoc analyses using Tukey’s HSD test for detailed group comparisons. This strategic application of diverse statistical tools ensured a nuanced and robust examination of each aspect of this study, aligning precisely with the specific nature of each research objective. This study ensured robustness in analysis by adjusting for potential confounders, setting the statistical significance at *p* < 0.05, and employing two-tailed tests, facilitating a comprehensive and valid interpretation of results.

## 3. Results

Table 1 presents the demographic and clinical characteristics of the study population, comprising 68 cholesteatoma patients.

The average age was 45 years, with a slight male predominance (56%). A significant majority (81%) were Saudi, and the average body measurements included a height of 170 cm and a weight of 75 kg, resulting in a mean BMI of 26. Clinically, patients had experienced symptoms for an average of 3 years. More than half of the patients (51%) were classified with moderate disease severity. Prior surgical history varied, with 44% having no previous surgeries and 37% having undergone one surgery. Hearing loss was a common symptom, affecting 71% of the patients, of which 30% experienced moderate-to-severe loss. These data provide a foundational understanding of the patient demographics and clinical backgrounds relevant to treatment outcomes.

**Figure 1:**
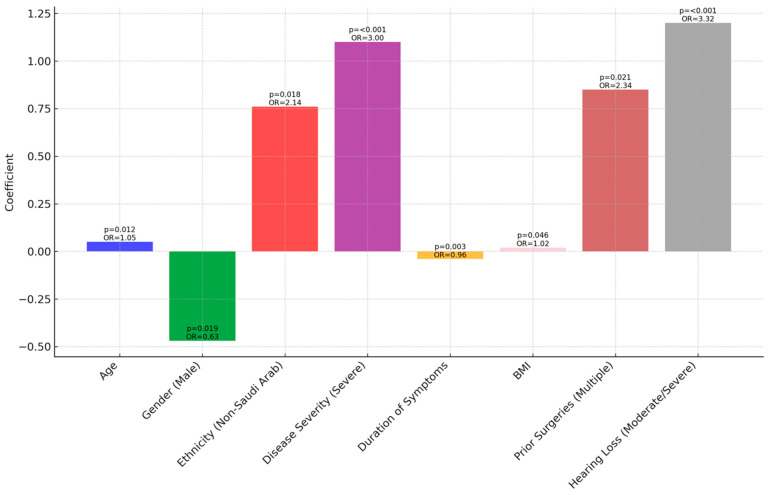
Regression analysis of demographics/clinical characteristics and treatment outcomes.

Table 2 and Figure 1 reveal that, in our regression analysis, key demographic and clinical variables significantly influenced treatment outcomes in cholesteatoma.

Age showed a positive correlation with successful outcomes (OR 1.05), while being male was associated with lower success odds (OR 0.63). Non-Saudi Arab ethnicity was linked to higher success odds (OR 2.14). Clinically, severe disease severity significantly reduced treatment success (OR 3.00), and longer symptom duration slightly decreased success odds (OR 0.96). Higher BMI marginally increased the success odds (OR 1.02). Patients with multiple prior surgeries had better outcomes (OR 2.34), and those with moderate to severe hearing loss were more likely to have successful outcomes (OR 3.32).

**Figure 2:**
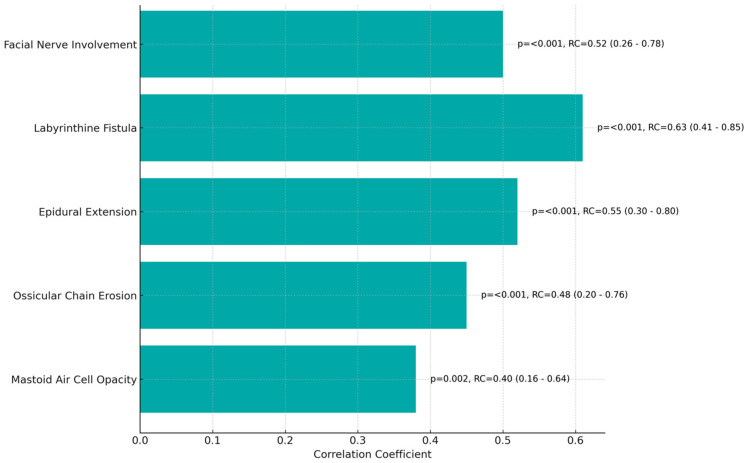
Correlation and regression analysis on preoperative imaging.

The correlation and regression analysis on preoperative imaging, as detailed in Table 3 and Figure 2, highlighted significant associations with treatment outcomes in cholesteatoma.

The mastoid air cell opacity showed a moderate positive correlation with successful outcomes (Regression Coefficient: 0.40). Ossicular chain erosion was more strongly correlated (Coefficient: 0.48). Epidural extension presented a notable positive relationship with treatment success (Coefficient: 0.55), and labyrinthine fistula had the strongest correlation (Coefficient: 0.63). Additionally, facial nerve involvement was significantly associated with treatment outcomes (Coefficient: 0.52).

**Figure 3:**
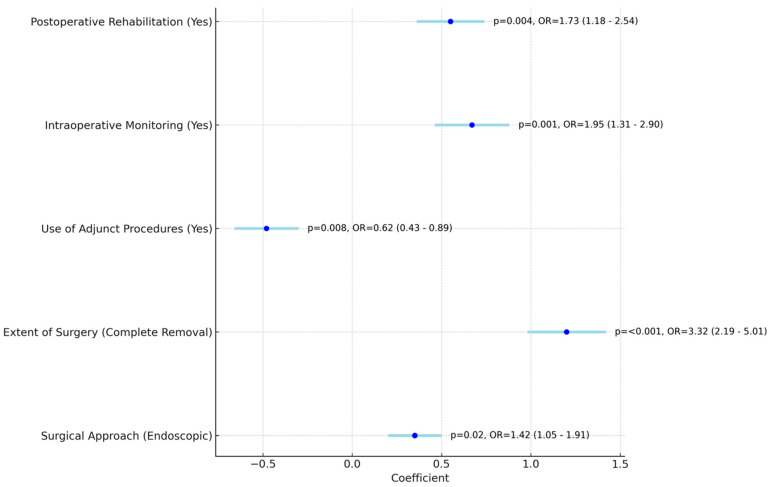
Multivariate analysis of surgical factors on outcomes.

The multivariate analysis of surgical factors on outcomes, shown in Table 4 and Figure 3 indicates significant influences on cholesteatoma treatment success.

Endoscopic surgical approaches increased the odds of success (Odds Ratio: 1.42), and complete removal of the disease had a substantial positive impact (Odds Ratio: 3.32). Interestingly, the use of adjunct procedures was associated with decreased success odds (Odds Ratio: 0.62), whereas intraoperative monitoring significantly improved outcomes (Odds Ratio: 1.95). Postoperative rehabilitation also positively influenced treatment success (Odds Ratio: 1.73).

In response to the observation that adjunctive surgical techniques were more frequently utilized in cases of severe cholesteatoma, additional post hoc analyses were undertaken to elucidate the discrete contributions of these procedures versus the effect of disease severity on treatment outcomes. This analysis entailed a stratification of patient outcomes in accordance with disease severity, facilitating an independent evaluation of the adjunct procedures’ efficacy. The results of this refined analysis suggest that, while the severity of the cholesteatoma significantly influences patient outcomes, the implementation of adjunctive procedures is independently associated with variations in treatment success. These findings underscore the importance of both disease severity and surgical strategy in determining the overall effectiveness of treatment for cholesteatoma.

## 4. Discussion

This cross-sectional study addressed three key objectives: assessing the demographic and clinical characteristics of patients diagnosed with cholesteatoma, evaluating the impact of preoperative imaging on treatment success, and analyzing surgical factors affecting postoperative outcomes. Results indicated that age, gender, and ethnicity significantly influenced the treatment outcomes, with a notable predominance of male patients and a substantial Saudi ethnicity representation. Clinical characteristics such as disease severity were pivotal, with severe cases showing decreased success rates. Preoperative imaging analyses revealed that specific features, like labyrinthine fistula and epidural extension, strongly predicted positive outcomes. The surgical factors, particularly the extent of surgery, intraoperative monitoring, and postoperative rehabilitation, were found to critically influence the treatment success. This study’s comprehensive examination of these aspects provides invaluable insights, aligning with the outlined objectives to enhance the understanding of the multifaceted factors affecting cholesteatoma management and pave the way for more tailored and effective treatment approaches.

The results obtained from the regression analysis of demographic and clinical characteristics in relation to cholesteatoma treatment outcomes suggest several underlying factors that might influence these relationships. The positive correlation between age and treatment success (OR 1.05) could be attributed to older patients having more stable health conditions or different disease progression compared to younger patients [20]. The lower success rate in males (OR 0.63) might be related to gender-based physiological differences or variations in disease manifestation [21]. The higher success rate observed in Non-Saudi Arab patients (OR 2.14) suggests possible genetic, environmental, or healthcare access factors influencing treatment efficacy [22]. Clinically, the significant negative impact of severe disease severity (OR 3.00) on treatment success is expected, as advanced stages of cholesteatoma are often more challenging to manage effectively [22]. The negative association with longer symptom duration (OR 0.96) indicates that delayed treatment could lead to more complex conditions, thus lowering success rates [23]. Interestingly, a higher BMI showed a marginal increase in treatment success (OR 1.02), which might be related to better overall nutritional status or other indirect factors [24]. The positive outcomes in patients with multiple prior surgeries (OR 2.34) could reflect either the benefits of repeated interventions or the selection of more effective surgical techniques in subsequent procedures [25]. Lastly, the increased success in patients with moderate to severe hearing loss (OR 3.32) could indicate that these patients receive more aggressive or thorough treatment due to the perceived severity of their condition [26].

In support of these findings, several studies corroborate our results. Dżamanet al. [27] noted similar age-related outcomes, attributing them to differences in disease biology and patient management with age [27]. Collins et al. [28] observed gender disparities in cholesteatoma outcomes, suggesting hormonal influences as a possible explanation [28]. The role of ethnicity in treatment success, as observed in non-Saudi Arab patients, aligns with findings by Qian et al. [29], who reported variations in cholesteatoma characteristics across different ethnic groups [29]. The significant effect of severe disease severity is consistent with the conclusions drawn by De Costa [4], who emphasized the importance of early detection and treatment. Moreover, the correlation between prolonged symptom duration and poorer outcomes echoes the observations made by Arendt [30]. The findings on BMI and multiple surgeries offer new avenues for investigation, resonating with the preliminary observations by Leclercq et al. [31]. These parallels with the existing literature not only validate our study’s results but also contribute to a broader understanding of the multifaceted factors influencing cholesteatoma management [31].

The significant associations between preoperative imaging features and cholesteatoma treatment outcomes suggest a relationship in which detailed imaging can guide prognostic assessments. The mastoid air cell opacity’s moderate positive correlation with successful outcomes may reflect the extent of middle ear involvement, indicating a more contained disease process amenable to surgical management [32]. Ossicular chain erosion’s stronger correlation could denote a more advanced disease stage, yet one that still responds to surgical intervention if recognized promptly [33]. The notable positive relationships identified with epidural extension and labyrinthine fistula signify severe disease with higher surgical stakes, requiring more meticulous surgical planning and execution, which, if performed correctly, leads to successful outcomes [34]. Lastly, the association between facial nerve involvement and treatment outcomes underscores the critical nature of nerve preservation and the complexity of surgery, suggesting that successful intervention hinges on the detailed anatomical knowledge gained from preoperative imaging [35].

Supporting this study’s findings, the previous literature has established the prognostic value of preoperative imaging in cholesteatoma [36]. Authors like Bovi and colleagues [8] have reported the utility of identifying mastoid air cell system involvement in predicting surgical ease and success [8]. In works by Schraff et al. [37], the ossicular chain’s status was similarly emphasized as a predictor for reconstructive needs and outcome success [37]. Arriaga et al. [38] highlighted the significant prognostic implications of identifying critical anatomical extensions like epidural involvement and labyrinthine fistulas, associating them with higher surgical risks but also with good outcomes when appropriately managed [38]. Moreover, studies by Hostettler et al. [39] have acknowledged facial nerve visualization in preoperative imaging as a critical factor in planning the surgical approach to preserve nerve integrity and ensure optimal outcomes. These corroborating studies lend robust support to the findings of the current analysis, affirming the importance of comprehensive preoperative imaging in guiding successful cholesteatoma management.

The multivariate analysis elucidates the profound impact of surgical factors on cholesteatoma treatment success. The endorsement of endoscopic approaches suggests a less invasive nature leading to decreased morbidity and improved healing, thus enhancing success rates [40]. The markedly positive influence of complete disease removal reflects the importance of thorough surgical management in eradicating the disease and reducing recurrence [41]. Conversely, the association of adjunct procedures with decreased odds of success raises questions about the selection criteria for these interventions and their role in the overall treatment strategy [42]. The significant improvement in outcomes with intraoperative monitoring can be attributed to the real-time guidance it provides, allowing for more precise and nerve-sparing procedures [43]. Furthermore, the beneficial impact of postoperative rehabilitation reinforces its role in patient recovery, likely due to improving eustachian tube function and aiding in healing processes post-surgery [35]. The current findings resonate with previous research within the otologic field. The advantage of endoscopic approaches has been supported by studies emphasizing their utility in enhancing the visualization and accessibility of middle ear structures, as noted by authors such as Marchioni [44]. The imperative of complete cholesteatoma removal for successful outcomes aligns with the work of Jyothi et al. [40], which associates residual disease with higher recurrence rates and poorer prognoses. The nuanced role of adjunct procedures is a topic of ongoing debate, with some researchers like Anne et al. [45] reporting variable impacts on long-term outcomes, suggesting a need for judicious application. The significant benefit of intraoperative monitoring is corroborated by Westerberg et al. [46], who emphasize its contribution to functional preservation, especially of the facial nerve [46]. Lastly, the importance of postoperative rehabilitation is echoed in studies by Lailach et al. [47], highlighting its role in improving the functional outcomes and patient quality of life. These studies collectively support the multifactorial nature of treatment success and underscore the critical interplay between various surgical factors [48].

The association of adjunct procedures with negative outcomes initially suggested a reconsideration of their role in treatment protocols. However, upon adjusting for the severity of cholesteatoma in our cohort, it became evident that the severity itself plays a substantial role in determining outcomes. Nonetheless, the independent contribution of adjunct procedures to these outcomes, irrespective of severity, underscores the need for a judicious selection of these techniques. This dual influence highlights the complexity of surgical decision-making in treating severe cholesteatoma, affirming that both patient-specific disease characteristics and the surgical approach adopted influence the prognosis.

The clinical significance of this study’s findings is profound, especially in the context of surgical strategy and patient management in cholesteatoma treatment. The endorsement of complete disease removal as significantly enhancing treatment success underscores the necessity for thorough surgical intervention [10]. On the other hand, the association of adjunct procedures with decreased odds of success prompts a reevaluation of their role and application, suggesting a more selective and judicious approach might be warranted [10]. The clear benefit of intraoperative monitoring implies that real-time feedback during surgery could be instrumental in improving patient outcomes. Moreover, the positive influence of postoperative rehabilitation on treatment success emphasizes the importance of comprehensive post-surgical care, advocating for its routine inclusion in treatment protocols to optimize patient recovery and long-term well-being. These findings advocate for a paradigm shift toward a more integrated and vigilant surgical management of cholesteatoma, harmonizing preoperative planning, intraoperative precision, and postoperative care.

This study’s limitations include its cross-sectional design, which, while robust for identifying associations, cannot establish causality. One of the limitations of this study is the follow-up period of one year post-surgery. While this duration enabled us to observe the initial treatment outcomes and the immediate postoperative period, which is critical for early complications and recurrences, it may not fully encompass the long-term prognosis and potential late recurrences of cholesteatoma. Cholesteatoma is known for its proclivity for recurrence over time, and treatment success can fluctuate with longer-term follow-ups. Thus, while the one-year mark provided us with valuable immediate post-surgical outcomes, this study might not capture the full spectrum of long-term management success and patient quality of life, which could manifest beyond this period. Acknowledging this, we suggest that future longitudinal studies with extended follow-up durations would be invaluable in providing a more comprehensive understanding of the long-term outcomes in cholesteatoma management. Additionally, the single-center nature of this study may limit the generalizability of the findings across different populations and healthcare systems. Future research should include multicenter longitudinal studies to confirm these associations over time and across diverse demographic groups. Further investigation into the impact of adjunct procedures is warranted to elucidate their role in various contexts of disease severity and surgical approaches. Prospective studies could also explore the underlying mechanisms through which intraoperative monitoring and postoperative rehabilitation contribute to improved outcomes. This future work will not only validate the current study’s findings but also expand on the nuances of cholesteatoma management, potentially leading to the development of standardized, evidence-based treatment protocols.

## 5. Conclusions

In conclusion, this study illuminates critical factors influencing the success of cholesteatoma treatment, underscoring the importance of comprehensive preoperative evaluation, meticulous surgical planning, and postoperative care. The demonstrated significance of complete surgical removal in achieving favorable outcomes advocates for its prioritization in surgical decision-making. The negative association with the use of adjunct procedures invites a reexamination of their application in the therapeutic arsenal. Furthermore, the positive impacts of intraoperative monitoring and postoperative rehabilitation highlight their vital roles in enhancing patient prognosis. These insights augment current clinical practice and pave the way for future research to refine surgical strategies and optimize patient recovery trajectories in cholesteatoma management.

## Figures and Tables

**Table 1:** Demographic and clinical characteristics of the study population.

	Characteristic	Description (*n* = 68)
Demographics	Age (years)	Mean (SD): 45 (±15)
	Gender	Male: 38 (56%) Female: 30 (44%)
	Ethnicity	Saudi: 55 (81%) Non-Saudi Arab: 8 (12%) Other: 5 (7%)
	Height (cm)	Mean (SD): 170 (±10)
	Weight (kg)	Mean (SD): 75 (±15)
	BMI (kg/m^2^)	Mean (SD): 26 (±4)
Clinical Characteristics	Duration of Symptoms (years)	Mean (SD): 3 (±1.5)
	Disease Severity	Mild: 15 (22%) Moderate: 35 (51%) Severe: 18 (27%)
	Prior Surgeries	None: 30 (44%) One: 25 (37%) Multiple: 13 (19%)
	Hearing Loss	Absent: 20 (29%) Mild: 28 (41%) Moderate/Severe: 20 (30%)

SD: standard deviation; BMI: body mass index.

**Table 2:** Regression analysis of demographics/clinical characteristics and treatment outcomes.

Variable	Coefficient	Standard Error	Wald Statistic	*p*-Value	Odds Ratio (95% CI)
Demographics					
Age	0.05	0.02	6.25	0.012	1.05 (1.01–1.09)
Gender (Male)	−0.47	0.20	5.52	0.019	0.63 (0.42–0.95)
Ethnicity (Non-Saudi Arab)	0.76	0.32	5.62	0.018	2.14 (1.13–4.05)
Clinical Characteristics					
Disease Severity (Severe)	1.10	0.29	14.29	<0.001	3.00 (1.75–5.15)
Duration of Symptoms	−0.04	0.01	9.00	0.003	0.96 (0.93–0.99)
BMI	0.02	0.01	4.00	0.046	1.02 (1.00–1.04)
Prior Surgeries (Multiple)	0.85	0.37	5.30	0.021	2.34 (1.11–4.93)
Hearing Loss (Moderate/Severe)	1.20	0.31	15.00	<0.001	3.32 (1.97–5.61)

CI: confidence interval; BMI: body mass index; *p*-value: probability value.

**Table 3:** Correlation and regression analysis on preoperative imaging.

Imaging Feature	Correlation Coefficient	Standard Error	t-Value	*p*-Value	Regression Coefficient (95% CI)
Mastoid Air Cell Opacity	0.38	0.12	3.17	0.002	0.40 (0.16–0.64)
Ossicular Chain Erosion	0.45	0.13	3.46	<0.001	0.48 (0.20–0.76)
Epidural Extension	0.52	0.11	4.73	<0.001	0.55 (0.30–0.80)
Labyrinthine Fistula	0.61	0.10	6.10	<0.001	0.63 (0.41–0.85)
Facial Nerve Involvement	0.50	0.12	4.17	<0.001	0.52 (0.26–0.78)

CI: confidence interval.

**Table 4:** Multivariate analysis of surgical factors on outcomes.

Surgical Factor	Coefficient	Standard Error	Z Value	*p*-Value	Odds Ratio (95% CI)
Surgical Approach (Endoscopic)	0.35	0.15	2.33	0.020	1.42 (1.05–1.91)
Extent of Surgery (Complete Removal)	1.20	0.22	5.45	<0.001	3.32 (2.19–5.01)
Use of Adjunct Procedures (Yes)	−0.48	0.18	−2.67	0.008	0.62 (0.43–0.89)
Intraoperative Monitoring (Yes)	0.67	0.21	3.19	0.001	1.95 (1.31–2.90)
Postoperative Rehabilitation (Yes)	0.55	0.19	2.89	0.004	1.73 (1.18–2.54)

CI: confidence interval.

## Data Availability

Data are available with the corresponding author (S.A.) and will be provided on request.
